# Challenges and Solutions in Recruiting Older Vulnerable Adults in Research

**DOI:** 10.3389/ijph.2024.1607247

**Published:** 2024-07-30

**Authors:** Nadia Sourial, Jean-Baptiste Beuscart, Łukasz Posłuszny, Matthieu Calafiore, Sónia S. Sousa, Esther Sansone, Marcelina Zuber, Isabelle Vedel, COVERAGE Collaborative Group

**Affiliations:** ^1^ Department of Health Management, Evaluation and Policy, School of Public Health, University of Montreal, Montreal, QC, Canada; ^2^ The University of Montreal Hospital Research Centre (CRCHUM), Montreal, QC, Canada; ^3^ Faculty of Medicine, Centre Hospitalier Universitaire Lille, University of Lille, Lille, France; ^4^ Department of Sociology, University of Wroclaw, Wroclaw, Poland; ^5^ Department of Family Medicine, University of Lille, Lille, France; ^6^ Psychological Neuroscience Laboratory, CIPsi, School of Psychology, University of Minho, Braga, Portugal; ^7^ Espace de Réflexion Éthique Régional des Hauts-de-France, University of Lille, Lille, France; ^8^ Department of Sociology, University of Wrocław, Wroclaw, Poland; ^9^ Department of Family Medicine, McGill University, Montreal, QC, Canada; ^10^ Lady Davis Institute, Jewish General Hospital, Montreal, QC, Canada

**Keywords:** recruitment, challenges, older adults, vulnerability, equity

## Introduction

Older vulnerable adults (OVA) are among the populations that interact the most with the healthcare system. Vulnerability in older adults has been broadly defined as the combination of frailty, “a clinically recognizable state in which older people’s ability to cope with daily or acute stressors,” and precarity, which “refers to insecurities and risks in the context of economic and social change, the hazards of contemporary life” [1].

Recruiting OVA can present particular challenges given their physical, cognitive, social (cultural, socioeconomic, language barriers) and political vulnerabilities [2] and are often excluded from research [3–5]. While general recommendations exist in addressing recruitment challenges in older adults [2, 6] and in hard-to-reach populations [7], there is a lack of methodological guidance specific to the recruitment of OVA whether it be in the context of quantitative or qualitative research. We collaborated with 26 international experts and researchers in aging from five countries (France, Italy, Poland, Lithuania, Portugal, Canada), part of an international research consortium on the care experiences of OVA [1], to highlight evidence-based solutions to meet these challenges. Consultations with experts were conducted through 27 workshops, including three 2-day in-person meetings organized Vulnerâge chair at the University of Lille and 24 virtual meetings between 2021 and 2023. A range of research projects involving the recruitment of OVA was discussed including a mix of quantitative (e.g., observational, quasi-experimental) and qualitative studies (e.g., descriptive, analytical). We chose to focus and highlight common challenges across these designs and across countries. A core group from the consortium, who are main authors of this article, synthesized the common challenges and proposed evidence-based solutions. This preliminary set of recommendations was then sent to the broader consortium for revision and validation. This manuscript includes an overview of three specific challenges and proposed solutions for the recruitment of OVA in research: finding the right recruitment process to foster representativeness, facilitating informed consent and promoting retention. A table of recommandations is also provided for easy use (Table 1).

**TABLE 1:** Recommendations to facilitate the recruitment of older vulnerable adults in research based on best practices and COVERAGE Collaborative Group discussion meetings in Lille, France, year 2023–2024.

1. Enhance representativeness o Focus on recruiting a diverse range of participants, including those from socially isolated or marginalized groups, to ensure findings are generalizable and applicable to all segments of the population. o Ensure eligibility criteria align with research objectives rather than ease of access
2. Develop Age-Friendly Recruitment Materials o Create recruitment materials that are simple, concise, and age-appropriate. These should be co-designed with stakeholders to ensure they meet the specific needs of older vulnerable adults. o Use multiple modes of communication, such as newspapers, online social media, mass media, and local venues frequented by older adults (e.g., primary care clinics, churches) o Utilize face-to-face contact, word-of-mouth, and trusted healthcare professionals to reach hard-to-reach populations, thereby fostering trust and building strong connections with targeted communities
3. Engage Trusted Influencers o Leverage peers and community influencers who can share positive experiences of participating in research to build trust and encourage participation among older vulnerable adults. o Involve caregivers, healthcare professionals, and social workers in the recruitment process to legitimize and facilitate study acceptance
4. Adapt the consent process o Conduct initial consent discussions in safe, familiar environments like the patient’s home to create a relaxed atmosphere. o Favor verbal consent over written consent when possible, and ensure information is conveyed in plain language, with adjustments for auditory limitations (e.g., speaking loudly and slowly) o Use tools to assess the capacity to consent individually and avoid blanket exclusions of those with cognitive impairments
5. Support Training and Education o Offer training programs for researchers and healthcare professionals on age-friendly communication techniques, ethical considerations in recruiting older vulnerable adults, and strategies for obtaining informed consent from individuals with cognitive impairments. o Share best practices, methodological innovations, and evidence-based solutions for recruiting older vulnerable adults in different contexts
6. Promote retention of participants o Design studies that provide tangible benefits to participants, such as opportunities for socialization and access to test results, while minimizing the need for travel and simplifying procedures. o Tailor follow-up methods to individual preferences and needs and offer flexible scheduling options to accommodate participants' availability o Maintain regular contact with participants through phone calls, newsletters, and greeting cards to keep them engaged and remind them of the importance of their participation

### Finding the Right Recruitment Process to Foster Representativeness

It is usually of interest to take into account OVA’s diversity (e.g., very old age, socially isolated, rural, ethnicity, languages, education), type of care (e.g., home care) in order to explore a wide range of experiences or needs [5, 8] It may be tempting to exclude hard-to-reach populations such as highly vulnerable and marginalized OVA (e.g., homeless, cognitively impaired). However, to foster representativeness, it is crucial that recruitment focus on the target population, eligibility criteria and research objectives [6] rather than easiness to reach.

Providing clear and understandable information about the study which is accessible to all OVA is another critical issue during the recruitment phase given their health and social vulnerabilities [6]. Recruitment requires an “age friendly” approach by recruiters, in which communication should be appropriately adapted to the needs of OVA [9] including the content, style, and approach [6]. In addition, caregivers and healthcare professionals, staff (e.g., nursing homes) might resist recruiting more vulnerable older patients [2].

Several solutions have been proposed in the literature [9–11]. For instance, messages should be age-appropriate and drafted with stakeholders [11], and need to be in short and explicit paper format [2, 10]. Multiple modes of communication should also be considered, such as newspapers, online social media, and mass media that have their own benefits and risks. For example, recruiting OVA through mass email lists will be insufficient to capture the segment of OVA that are less likely to participate or who do not have access to technology [10]. The recruitment strategy should also focus on local venues where older people usually go (e.g., primary care clinics, church) [5]. Peers may also be leveraged as influencers and can share their positive perceptions in participating in research [9]. Finally, recruitment of hard-to-reach populations is most efficient if done by face-to-face contact, word-of-mouth, families or by known and trusted healthcare professional [5, 10] in order to convey reassurance, trustworthiness and develop strong connections with targeted communities. It is thus key to involve and educate resource people (healthcare professionals, social workers, relatives, etc.) in the field in order to help facilitate and legitimize acceptance of the study [9, 11].

### Facilitating Informed Consent

Informed consent is particularly difficult for OVA as it strongly influences social relationships and can feel threatening for individuals who may fear losing health services if they refuse to participate [12]. In addition, ensuring informed consent for very vulnerable OVA such as those with cognitive disorders is particularly challenging [13]. Blanket exclusion of those with cognitive impairment is to be avoided [6]. Instead, decisions regarding the capacity to consent should be based on an individual basis [6].

The literature proposes several potential solutions to address challenges which are specific to OVA [13]. First and foremost, first encounters by researchers should ideally happen in a safe place and relaxed atmosphere such as the patient’s home [11]. When possible, verbal consent should be favored over signed consent which tends to generate more concerns among OVA [12]. In addition to ensuring information is conveyed in an appropriate and plain language, care should be taken to speak loudly and slowly where patients have auditory limitations [2]. Time should be taken to explain and repeat the purpose of the study, how the study will be conducted and how results will be kept safe and confidential [2]. Informed consent for OVA with cognitive impairment is particularly challenging [14, 15]. It is important to note that informed consent can often still be obtained for persons at mild and moderate stages of dementia while, consent by a legal representative may be considered for more advanced stages to ensure that the decision to participate or not is based on the person’s wishes and preferences. Using tools to assess the capacity to consent is therefore an essential component in recruiting OVA for research [14, 15].

### Promoting Retention

Retention is usually an important challenge for longitudinal studies with OVA who are prone to fatigue, cognitive impairment, disabilities, illness or sensory limitations [2, 16]. Research teams should plan a variety of strategies to facilitate recruitment and retention. Designing studies that maximises the benefit/burden ratio can help with recruitment and retention [16] such as providing opportunity for socialization, access to test results [2, 16] and minimizing travel [16, 17]. Finally, regular “keep in touch” calls and mailings (i.e., newsletters and greeting cards) have been shown to increase retention [17].

## Conclusion

In conclusion, recruitment methods need to be adapted to consider the particular physical, mental and social aspects of OVA. In this context, it is key to reflect on these challenges inherent to the recruitment and consenting process to promote representativeness and retention. Table 1 proposes a summary of the recommendations to address these challenges. These recommendations demonstrate the need to consider and incorporate a variety of methods to help ensure that recruitment is adapted to OVA’s needs and reality, leading to richer and more meaningful research results. While this study focused on recruitment challenges common across designs and jurisdictions, future research could consider a more in-depth analysis of design-specific challenges and contextual differences between countries given the different legal frameworks, healthcare systems, and research infrastructure.
